# Maturation Pathways of Cross-Reactive HIV-1 Neutralizing Antibodies

**DOI:** 10.3390/v1030802

**Published:** 2009-11-06

**Authors:** Xiaodong Xiao, Weizao Chen, Yang Feng, Dimiter S. Dimitrov

**Affiliations:** Protein Interactions Group, Center for Cancer Research Nanobiology Program, National Cancer Institute, National Institutes of Health, Frederick, MD 21702, USA

**Keywords:** germline, antibody, immune responses, HIV, escape, vaccine

## Abstract

Several human monoclonal antibodies (hmAbs) and antibody fragments, including the best characterized in terms of structure-function b12 and Fab X5, exhibit relatively potent and broad HIV-1 neutralizing activity. However, the elicitation of b12 or b12-like antibodies *in vivo* by vaccine immunogens based on the HIV-1 envelope glycoprotein (Env) has not been successful. B12 is highly divergent from the closest corresponding germline antibody while X5 is less divergent. We have hypothesized that the relatively high degree of specific somatic hypermutations may preclude binding of the HIV-1 envelope glycoprotein (Env) to closest germline antibodies, and that identifying antibodies that are intermediates in the pathways to maturation could help design novel vaccine immunogens to guide the immune system for their enhanced elicitation. In support of this hypothesis we have previously found that a germline-like b12 (monovalent and bivalent scFv as an Fc fusion protein or IgG) lacks measurable binding to an Env as measured by ELISA with a sensitivity in the μM range [1]; here we present evidence confirming and expanding these findings for a panel of Envs. In contrast, a germline-like scFv X5 bound Env with high (nM) affinity. To begin to explore the maturation pathways of these antibodies we identified several possible b12 intermediate antibodies and tested their neutralizing activity. These intermediate antibodies neutralized only some HIV-1 isolates and with relatively weak potency. In contrast, germline-like scFv X5 neutralized a subset of the tested HIV-1 isolates with comparable efficiencies to that of the mature X5. These results could help explain the relatively high immunogenicity of the coreceptor binding site on gp120 and the abundance of CD4-induced (CD4i) antibodies in HIV-1-infected patients (X5 is a CD4i antibody) as well as the maturation pathway of X5. They also can help identify antigens that can bind specifically to b12 germline and intermediate antibodies that together with Envs could be used as a conceptually novel type of candidate vaccines. Such candidate vaccines based on two or more immunogens could help guiding the immune system through complex maturation pathways for elicitation of antibodies that are similar or identical to antibodies with known properties.

## 1. Introduction

Elicitation of broadly cross-reactive HIV-1 neutralizing antibodies (bnAbs) *in vivo* is rare. It is thought that this is likely due to protection of conserved structures of the virus envelope glycoprotein (Env) by variable loops, extensive glycosylation, occlusion within the oligomer, and conformational masking, and the rapid generation of HIV-1 mutants that outpace the development of such antibodies [2–5]. A number of Env-specific hmAbs have been identified [6] but only several exhibit neutralizing activity to primary isolates from different clades [4,7] including IgG b12 [8,9], IgG 2G12 [10–12], m14 [13], m18 [14], 447–52D [15], IgG 2F5 [16], IgG 4E10 [17,18], IgG m46 [19], IgG m48 [20], Fab X5 [21] and Fab Z13 [18]. Of those b12, 2G12, 2F5, 4E10 are best characterized and considered to exhibit on average the broadest and most potent neutralizing activity. X5 exhibits comparable or even more potent and broad neutralizing activity which however is dependent on size – the smallest fragment (scFv) is the most potent followed by Fab and IgG [22]. The full-size X5 antibody in the IgG1 format is significantly less potent although it can still neutralize some isolates. The existence of bnAbs such as b12 has fueled the hope that the development of efficacious HIV vaccine is achievable provided that an immunogen containing the epitopes of these antibodies is appropriately designed. However, in spite of the tremendous amount of research, the goal of an antibody-based effective vaccine based on appropriately designed and exposed or empirically found vaccine immunogen has not been achieved [23]. Our inability to achieve elicitation of such bnAbs in humans and the very low frequency of HIV-infected humans with potent bnAbs strongly suggest that there are still unknown fundamental immunological mechanisms that allow HIV to evade elicitation of bnAbs. Understanding these mechanisms could provide novel tools for development of efficacious vaccines.

We have previously analyzed the sequences of all known bnAbs and have found that they are highly divergent from germline antibodies [1,24]. B12 is especially highly somatically hypermutated while X5 is relatively less divergent from germline antibodies. We have hypothesized that the relatively high degree of specific somatic hypermutations may preclude binding of the HIV-1 envelope glycoprotein (Env) to closest germline antibodies, and that identifying antibodies that are intermediates in the pathways to maturation could help design novel vaccine immunogens to guide the immune system for their enhanced elicitation. In support of this hypothesis we have previously found that a germline-like b12 (monovalent and bivalent scFv as an Fc fusion protein or IgG) lacks measurable binding to an Env as measured by ELISA with a sensitivity in the μM range [1]. In contrast, a germline-like scFv X5 bound Env with high (nM) affinity.

Here we present evidence confirming and expanding these findings for a panel of Envs. We describe the identification of possible b12 intermediate antibodies that could serve as reagents for the identification of new vaccine immunogens that can help guide the immune system through the b12 maturation pathway.

## 2. Results

### 2.1. Binding of mature and germline-like scFv X5 to Env - dominant role of the heavy chain

**Figure 1.**
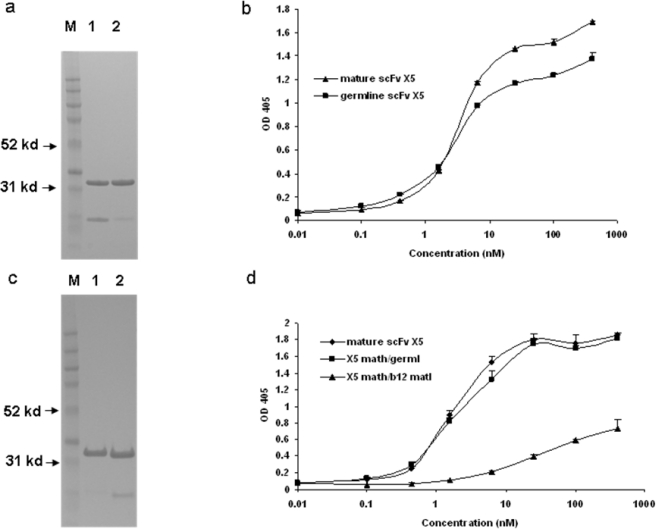
Binding characteristics of the scFv X5 in various forms including mature, germline (in this and subsequent figures the term germline is for convenience the representation of germline-like antibody in this study), and hybrids between various heavy and light chains. a and c. Gel analysis of purified mature, germline and hybrid scFv X5. In a, M, molecular weight marker, 1 and 2 are mature and germline scFv X5 respectively; In c, M, molecular weight marker, 1 is the hybrid scFv between mature X5 heavy chain and germline X5 light chain, and 2 is the hybrid between matured X5 heavy chain and matured b12 heavy chain. In b and d are bindings of purified proteins shown in a and c, respectively to bal gp120-CD4. Abbreviations in this and subsequenct figures are as following: math, matured heavy chain; germl, germline light chain; matl, matured light chain; and germh, germline heavy chain.

We have previously found that germline-like X5 binds to Env with relatively high strength (low EC50) which is only slightly lower than that for the mature antibody ((Figure 1) and [1]). To further characterize this interaction and explore the relative contributions of the heavy and light chains to the interaction we generated, expressed and purified hybrid molecules containing the heavy chain of X5 combined with the light chain of the germline-like X5 (Figure 1c). The hybrid between the mature X5 heavy chain and its germline-like light chain bound better than did the germline-like X5 but similarly to the mature X5, underlying the dominant role of the heavy chain in determining the binding specificity and affinity (Figure 1d). Since X5 and b12 share the same germline light chain V and J genes, another hybrid between mature X5 heavy chain and mature b12 light chain was generated. This hybrid bound weaker than the germline X5 (Figure 1d). This is unlikely due to improper protein folding since the soluble expression of this particular hybrid is comparable to that of mature, germline-like, and other hybrids of X5. In all cases the hybrid molecules bound the Env suggesting that the heavy chain of the mature X5 and likely of the germline-like X5 dominates the interaction (Figure 1).

### 2.2. Germline-like X5 neutralized a subset of pseudoviruses neutralized by the mature X5

**Figure 2.**
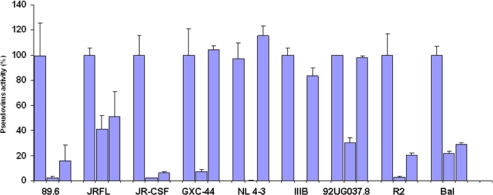
Inhibition of pesudovirus infection by mature and germline scFv X5. Nine HIV Env-pseudotyped viruses were tested with a single concentration of both X5 original and germline as described in Materials and Methods. The concentration of scFv used is 600 nM. The names of the Envs used are shown on the X-axis and the numbers on the Y-axis represent the percentages of the pseudovirus activities. For each isolate, the bars represent the percentage of activities of the viruses treated with PBS only (left columns), scFv X5 original (middle columns), and germline (right columns).

**Figure 3.**
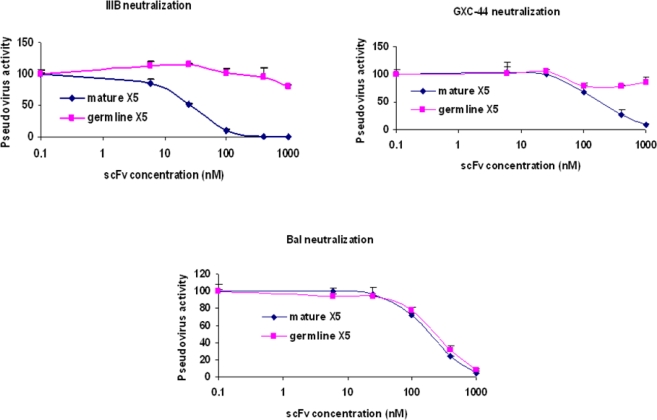
Determination of the IC50s of the mature and germline scFv X5 against representative HIV isolates. Viruses pseudotyped with Envs from M and T tropic viruses from B clade as well as one from A clade were used in the neutralization assay.

To test the neutralizing activity of germline-like scFv X5 relative to the mature antibody we used a panel of pseudoviruses with Envs from isolates from Clades A, B, and C. The mature X5 neutralized all of them efficiently at the concentration used. The germline-like X5 neutralized all B clade, R5 or dual tropic isolates almost as efficiently as the mature one, but lost completely the neutralizing ability against B clade X4 tropic isolates as well as isolates from other clades (Figure 2). To confirm this observation, dose response curves were constructed for both mature and germline-like X5 against three representative isolates (Figure 3). While the mature and germline-like X5 exhibited similar IC50s against Bal pseudovirus, there was complete lack of neutralization by the germline-like X5 against IIIB and GXC-44. These data, although based on a limited number of isolates tested, indicate a possible mechanism of how X5 could have evolved from a clade and tropism specific neutralizing antibody to a relatively broadly neutralizing antibody by acquiring somatic mutations. However, note that this activity is measured for X5 in a scFv format. As discussed above full-size X5 is less potent than scFv X5 and its maturation pathway *in vivo* is likely to be complex.

### 2.3. Lack of measurable binding of germline-like b12 as scFv and as a bivalent Fc fusion protein to a panel of Envs

**Figure 4.**
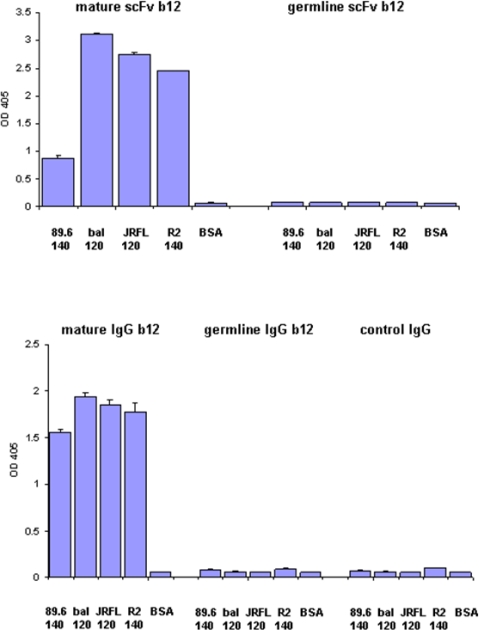
Lack of binding by germline scFv and IgG b12 to multiple HIV Envs. Mature and germline b12 in both scFv (upper panel) and IgG (lower panel) formats, and an irrelevant IgG were tested for their bindings to four different HIV Envs as indicated on X axis. BSA was included as a negative control. The single concentration used for each antibody was 2.5 μM.

We have previously found that germline-like b12 lacks measurable binding to an Env in our ELISA assay [1]. Here we confirm and extend this observation to a panel of Envs. In all cases we found that germline-like b12 in both formats, as a scFv and as a IgG lacks measurable binding to this panel of Envs (Figure 4). In contrast, as expected the mature b12 bound strongly to all tested Envs (Figure 4).

### 2.4. Identification of possible intermediates in the maturation pathway of b12

**Figure 5.**
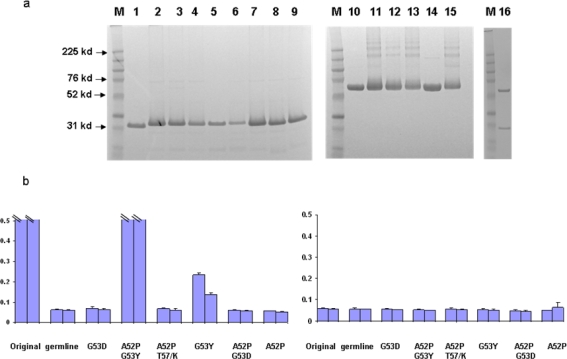
Identification of b12 intermediate binders. Point mutations were introduced back to the H2 and adjacent frame work of germline b12. The resulted mutants were expressed, purified both as scFv and scFv-Fc. a. Gel analysis of the purified scFv and scFv-Fc. M, molecular weight marker. Numbers on the left indicate molecular weight in kd. Samples 1–9 are original, germline, A52P/G53Y, G53Y, math/germl, germh/matl, G53D, A52P/T57K, and A52P. Samples 10–15 are mature b12-Fc, germline b12-Fc, A52P/G53Y-Fc, G53Y-Fc, math/germl-Fc, and germh/matl-Fc. Sample 16 is the germline IgG b12. b. The bindings by the selected scFv were analyzed against bal gp120 in an ELISA (left panel). Two concentrations including 8 (bar closer to Y-axis) and 2.7 μM of each scFv were used. BSA as an antigen was included as a specificity control (right panel). The maximum value of Y-axis, which shows OD 405 from ELISA assay was set at 0.5 to reflect the weak bindings. Bindings of both mature b12 and A52P/G53Y reached saturation at both concentrations and were indicated.

We began to identify possible b12 intermediates in its maturation pathway by introducing critical residues found in mature b12 back into the germline framework of b12. We started with H2 for several reasons. First, the heavy chain is likely the major determinant for binding as indicated by the crystal structure of b12 in complex with gp120. Secondly, most of the amino acid substitutions in H1 (4 of 5) between germline and mature forms are similar in nature. In contrast, all three mutations in H2 of the mature b12 resulted in amino acids that are very different from their germline counterparts. A series of mutants were generated surrounding the germline b12 H2 region as described in the Methods. In order to prevent potential poor expression or folding of certain mutants from distorting the data interpretation, all the mutants investigated were expressed and purified to homogeneity both as scFv and scFv-Fc soluble fusion proteins (Figure 5a). The b12 germline-like antibdy was also expressed in the IgG format (Fig 5a). In an initial screening with one single high antibody concentration, we found that a single mutation G53Y was sufficient to confer detectable binding ability to germline b12. Additional mutations, such as A52P, increased the binding ability significantly (Figure 5b).

**Figure 6.**
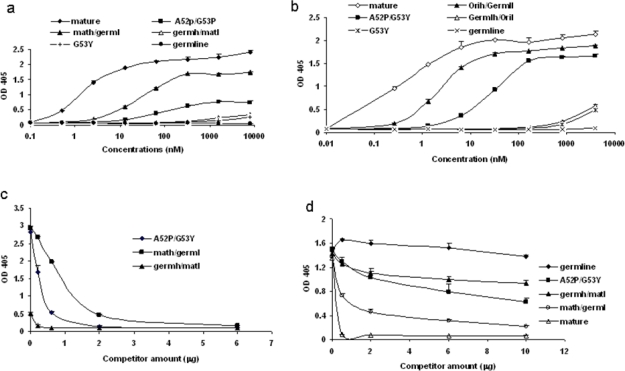
Determination of the strength of binding by various formats of b12. ELISAs were performed using bal gp120 as the antigen. Various scFv (a) and scFv-Fc (b) b12 as indicated were analyzed for their bindings. c. Competition ELISA between various scFv b12 and the original scFv-Fc b12. Fixed amount of various scFv b12 at 20 μg was pre-mixed with increasing amount of original scFv-Fc b12 in 100 μl of blocking buffer and applied to ELISA plate coated with bal gp120. The amount of bound scFv was measured using anti-his-HRP. d. Specific competition between sCD4 and various forms of b12 in binding to bal gp120. Fixed amount of sCD4 at 2 ug was mixed with increasing amount of various b12-Fc fusion protein in 100 μl of blocking buffer and added to ELISA plate coated with bal gp120. The bound sCD4 was detected with anti-his-HRP.

To confirm the data obtained in the initial screening with a single high concentration, we performed ELISA using a range of concentrations of various scFv and scFv-Fc b12. The binding by the germline mutant G53Y was consistently detectable (Figures 6a, b). Interestingly, a hybrid between mature b12 light chain and germline b12 heavy chain also displayed binding ability albeit weak (Figures 6a,b). The avidity effect was evident when the bindings by scFv and corresponding scFv-Fc were compared (Figures 6a, b). It is noteworthy that germline-like b12 consistently showed no binding even at the highest concentration. The binding characteristics of some of the intermediates are summarized in Table 1. We added two more layers of specificity control in addition to BSA, which was used as a control antigen in all experiments. First, we found that all the weak bindings detected by the b12 intermediates can be completely competed out by the mature b12 (Figure 6c). Further, all the b12 intermediates competed with sCD4 for binding to gp120, and the competition was proportional to their binding abilities. Germline-like b12, on the other hand, did not show any competition with sCD4 (Figure 6d). These results indicate that the two mutations, G53Y and A52P, could play a role in the pathway from germline b12 to mature b12.

### 2.5. Inhibition of pseudovirus infection by b12 intermediates

**Figure 7.**
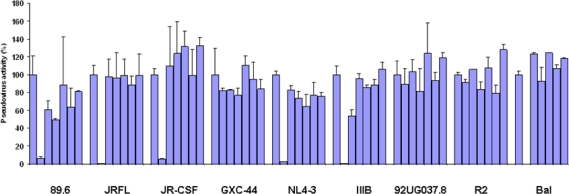
Inhibition of pseudovirus infection by various scFv-Fc b12. Nine HIV Env-pseudotyped viruses were tested with a panel of scFv-Fc b12 variants. The original-Fc b12 was used at a concentration of 0.3 μM, while all the other b12–Fc fusion variants including the germline-Fc b12 were used at a concentration of 2 μM. For each isolate, the bars represent the percentage of activities of the viruses treated with PBS only, b12-Fc, math/germl-Fc, A52P/G53Y-Fc, germh/matl-Fc, G53Y-Fc, and germline-Fc sequentially, with the PBS treated sample closet to the Y-axis.

The neutralizing abilities of mature, germline-like and various intermediate b12s in their scFv-Fc format were tested against a panel of HIV Env pseudotyped viruses. The mature b12-Fc neutralized efficiently all isolates from clade B except R2, which is a CD4 independent isolate. The mature b12-Fc also failed to inhibit two isolates from clade A and C including isolates GXC-44 and 92UG037.8. This is in agreement with previous findings that b12 is most efficient against B clade isolates [25]. None of the b12 intermediates displayed significant neutralizing ability with the exception of the hybrid mature heavy chain/germline light chain (math/germl), which has relatively high binding affinity (34.5 nM) but neutralized IIIB with modest activity at a concentration significantly higher than the concentration needed for 50% binding (Figure 7). These results indicate that some potential b12 intermediate antibodies may not exert selection pressure for generation of HIV-1 mutants.

### 2.6. Binding of germline-like and intermediate b12 antibodies to human cell lines

**Figure 8.**
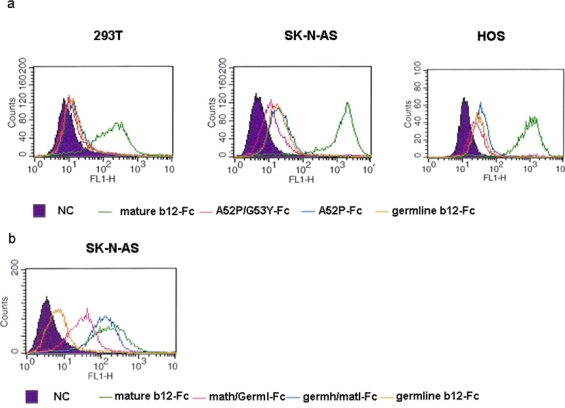
Binding of various b12-Fc proteins to the surface antigens of three human cell lines. (a). Germline-Fc, G53Y-Fc, A52P/G53Y-Fc and mature-Fc b12 proteins were used at a concentration of 1 μM in the flow cytometry assay as described. (b). Germline-Fc, math/germl-Fc, germh/matl-Fc and original-Fc were further compared in the flow cytometry assay. The concentration used remained at 1 μM. The numbers on the X-axis represent the binding intensity and the numbers on the Y-axis represent the number of cells.

The accumulating numbers of somatic mutations during the b12 maturation contributed to the incremental increase in its binding to Envs. To test how various b12 somatic mutations contribute to self antigen bindings we used three human cells lines in a flow cytometry analysis. We found that the mature b12 binds strongly to these cells (Figure 8) in agreement with previous data from Haynes group [26]. The germline-like b12 and intermediate b12 with small number of mutations (A52P/G53Y and A52/P) displayed much lower although measurable activities to these cell lines (Figure 8). Interestingly, when the intermediates with large number of mutations, such as hybrid math/germl and germh/matl antibodies were tested, they showed significantly higher human cell binding approaching the level displayed by the mature b12. These findings indicate that specific binding to Env and self antigens were probably acquired concomitantly through the b12 maturation process. In progress are experiments to determine the nature of the human molecules recognized by b12.

## 3. Discussion

Extensive somatic mutations have been found in all identified broadly neutralizing HIV antibodies and in most other HIV specific antibodies. This is in contrast to some of the potent neutralizing antibodies against acute infections [1,24,27–30]. These antibodies possess few if any mutations compared to their germline sequences. Our knowledge of whether germline-like antibodies, corresponding to the HIV-1-neutralizing antibodies, possess neutralizing activity and how the somatic mutations can contribute to their binding and neutralizing function is limited. To begin to increase our understanding of these and other properties of the antibody maturation pathways we analyzed the binding and neutralizing abilities of mature and germline forms of two HIV-1 neutralizing antibodies, X5 and b12.

IgG1 X5 is a modestly neutralzing antibody targeting a highly conserved CD4i epitope. Its corresponding germline antibody in a scFv format displayed high affinity to the Env tested in this study and neutralized efficiently several isolates. Intriguingly, all these neutralized isolates belong to B clade and are either R5 tropic or dual tropic. On the other hand, the mature scFv X5 neutralized all isolates tested. These isolates are from A, B and C clades. These data suggest first that germline antibodies against certain epitopes on the HIV-1 Env, similarly to antibodies against other acute infections, do possess neutralizing ability. Secondly, the mutational process shifted or expanded the antibody binding epitope so that it became more inclusive leading to a more broadly neutralizing antibody. This notion is supported by a previous observation that a synthetic HIV-1 inhibitor based on CH2, m1a1, has an epitope that partially overlaps with that of germline X5 as revealed by competition ELISA [1]. M1a1 has a tendency to neutralize only B clades, X4 tropic viruses in contrast to germline-like X5. The epitope of m1a1 overlaps significantly more with that of the mature X5 than with germline X5 as also revealed by competition ELISA [31].

Based on these data, one can speculate that X5 originated from a germline antibody recognizing a B clade, R5 tropic isolate. The subsequent mutations expanded its targets to include B clade, X4 tropic isolates and those from other clades. X5 seems to follow a typical antibody maturation pathway, and this might explain partially the predominant presence of CD4i antibodies in HIV patients due to the fact that this epitope appears to be readily available for germline antibody recognition. It remains to be seen if germline counterparts of other CD4i antibodies possess antigen binding and neutralizing abilities as observed with X5.

In contrast to X5 b12 appears to follow a different pathway. The germline b12 lacks observable binding to a panel of Envs confirming and expanding our original observation for lack of measurable binding of germline-like b12 to one Env [1]. By systematic mutation of amino acid residues in the mature b12 into the corresponding locations on germline b12, we identified several possible intermediates at different stages along the maturation pathway of b12. Importantly, the increase in binding against the Env associated with the increasing number of somatic mutations in these intermediates seems to be closely related to their increase in binding to human antigens. This revealed a potentially interesting interplay between the Env and human self antigens in the origination and maturation of b12. As was proposed in our previous study based on the lack of binding of germline b12 to HIV Envs, one or more alternative antigens, self-antigens included, were likely responsible for the initial activation of the B cells expressing b12 germline-like antibodies. The somatic mutations ensued after the activation may have enabled b12 intermediate(s) to bind to other antigens including Envs. The fact that a single mutation, G53Y, conferred detectable germline-like b12 binding to Env as found in this study indicates that this scenario is possible. Note however, similar to X5 b12 was selected through phage display procedure. Therefore the relevance of b12 as well as the intermediate binders identified in this study to *in vivo* situation remains hypothetical. These intermediate antibodies are currently used as reagents to identify molecules that could serve as primary immunogens for initiation of the maturation of b12 or b12-like antibodies. The primary immunogens to be found could be used in combination with appropriately designed Envs exposing the b12 epitopes and lacking other immunodominant epitopes. This conceptually new two (or more) immunogen approach for guiding the immune system through the complex maturation pathways of known antibodies with high activity is more general and could be used to help design of vaccine immunogens also for other diseases including cancer.

## 4. Materials and Methods

### 4.1. Primers, peptide and proteins

All the primers used in this study were purchased from Invitrogen (Carlsbad, CA). Bal gp120-CD4 was provided by Tim Fouts (University of Maryland, Baltimore, MD) and other recombinant proteins (gp120s and gp140s) were provided by Christopher Broder (USUHS, Bethesda, MD). Codon-optimized sCD4 D1-2 was cloned into the expression vector pSecTag2B (Invitrogen) attaching a His tag to the C terminus of the sCD4 D1-2, transfected into 293 freestyle cells and expressed according to the manufacturer’s suggested protocol. The secreted sCD4 D1-2 was purified using a Nickle column from the culture medium (Qiagen, Hilden, Germany).

### 4.2. Gene synthesis and expression plasmid constructions

ScFv DNAs corresponding to mature and germline-like X5 and b12 were synthesized by Genescript (Genescript, Piscatawy, NJ). The Vh of each of the antibodies was linked to the Vl via a (GGGGS)3 linker. The scFv fragment was cloned into the pComb3X plasmid (provided by Dennis Burton, Scripps Institute, La Jolla, Cal) for expression in bacteria. The DNA fragments encoding various b12 scFv antibodies were fused with Fc of human IgG1 and cloned into the mammalian cell expression vector pSecTag2B (Invitrogen) for expression of the scFv-Fc fusion proteins. The Vh and Vl of the germline b12 were further grafted to the pDR12 vector (provided by Dennis Burton) for conversion to IgG1 format.

### 4.3. Identification of intermediate affinity b12 binders

The degenerate primer B12H2 primer 5′ atg gga tgg atc aac Sct KRc aat ggt aac aMa aaa tat TCA CAG 3′ was used in an overlapping PCR to replace the residues at positions 52, 53, and 57 of the germline b12 H2 with corresponding residues from the b12 mature form. A collection of germline b12 variants containing one, two or three residues from mature b12 form were generated through this process.

### 4.4. Antibody expression and purification

For scFv expression, *E. coli* strain HB2151 was transformed by the X5 and b12 scFv constructs described above. Single clone was inoculated into 2YT supplemented with 100 units of ampicillin, 0.2 % glucose and incubated at 37°C with shaking. When the OD600 reached 0.9, IPTG was added to achieve a final concentration of 1 mM and the culture was continued with shaking for overnight at 30°C. Cells were then collected, lysed with polymyxin B (Sigma, St Louis) in PBS, and the supernatant was subjected to the Ni-NTA agarose bead (Qiagen) purification for the soluble scFvs. The various b12 scFv-Fc constructs as well as the germline b12 IgG construct were transfected into the 293 freestyle cells with polyfectin transfection agent (Invitrogen). Four days after transfection, the culture medium was collected and the secreted scFv-Fc and IgG proteins were purified using a protein-A sepharose column (GE Healthcare, Piscataway, NJ).

### 4.5. ELISA

Different protein antigens were diluted in the PBS buffer in concentrations ranging from 1–4 μg/mL and coated to the 96 well plate at 4°C for overnight. The plate was then blocked with PBS + 5% dry milk buffer. Antibodies in various formats were diluted in the same blocking buffer and applied to the ELISA plate. The mouse-anti-His-HRP was used to detect the His tag at the C terminus end of each of the scFvs in most of the ELISA and the mouse-anti-human Fc-HRP was used to detect the scFv-Fc and IgG bindings. ABTS was then added to each well and OD 405 was taken 5–10 minutes afterward. To estimate the EC50s of some of the binders, data generated from the dose response bindings were analyzed using the weighted least-squares fitting of data to the function (Hill equation).

### 4.6. Pseudovirus neutralization assay

HIV Env pseudotyped virus preparation and neutralization was performed as previously described.[32]

The activity of each pseudovirus isolate with or without antibody treatment was expressed as a percentage of the corresponding control virus without antibody treatment, and the average percentages and standard deviations were then calculated.

## Figures and Tables

**Table 1.** Summary of the binding characteristics of the mature, germline-like and intermediate scFv and scFv-Fc b12 as determined by ELISA. The antigen is bal gp120. DB, did not bind; N/A, did not test; “>μM”, binding affinity in the range above μM. Germh/matl, germline heavy chain fused with mature light chain. Math/germl, mature heavy chain fused with germline light chain.

**b12 construct**	**germline**	**A52P**	**A52P/T57K**	**G53Y**	**A52P/G53Y**	**germh/matl**	**math/germl**	**mature**
scFv	DB	DB	DB	>μM	87.3 nM	>μM	34.5 nM	1.3 nM
scFv-Fc	DB	N/A	N/A	>μM	29.3 nM	>μM	2.6 nM	0.4 nM
